# Factorial structure and measurement invariance of the Chinese version of the Oral Health Impact Profile-14 among clinical populations and non-clinical populations: an evidence for public oral investigations

**DOI:** 10.1186/s12903-023-03310-6

**Published:** 2023-08-24

**Authors:** Guang-Hui Yang, Yao Feng, Lan-Xin Xue, Ze-Yue Ou-Yang, Yi-Fan Yang, Ya-Qiong Zhao, Jie Zhao, Jing Hu, Qin Ye, Xiao-Lin Su, Ning-Xin Chen, Meng-Mei Zhong, Yun-Zhi Feng, Yue Guo

**Affiliations:** Department of Stomatology, The Second Xiangya Hospital of Central South University, Changsha, Hunan 410011 China

**Keywords:** Oral Health Impact Profile-14, Oral health-related quality of life, Measurement invariance, Confirmatory factor analysis, Oral investigations

## Abstract

**Objective:**

Oral health-related quality of life (OHRQoL) is a multidimensional concept that is commonly used to examine the impact of oral health status on quality of life. The purpose of this study was to examine the optimal factor model of the Chinese version of the Oral Health Impact Profile (OHIP-14) questionnaire in clinical populations, measurement invariance across clinical status and gender cohorts. This would ensure equal validity of the Chinese version of OHIP-14 in different populations and further support public oral investigations.

**Methods:**

The Chinese version of OHIP-14 was used to investigate 490 dental patients and 919 college students. Confirmatory factor analysis (CFA), item analysis and reliability, measurement invariance, and the t-test were used for data analyses.

**Results:**

We found that the 7-factor structure had the best-fit index in the sample (CFI = 0.970, TLI = 0.952; SRMR = 0.029, RMSEA = 0.052(0.040,0.063)). The reliability of the scales was satisfactory (Cronbach’s α = 0.942). The error variance invariance fitted the data adequately in measurement invariance, indicating that measurement invariance is acceptable both across the clinical and non-clinical populations (∆CFI=-0.017, ∆RMSEA = 0.010) and across genders in the clinical population (∆CFI = 0.000, ∆RMSEA=-0.003). T-test for scores showed that the clinical populations scored significantly higher than the non-clinical populations, as did the overall score (t = 7.046, *p* < 0.001, d = 0.396), in terms of functional limitation (t = 2.178, *p* = 0.030, d = 0.125), physical pain (t = 7.880, *p* < 0.001,d = 0.436), psychological discomfort (t = 8.993, *p* < 0.001, d = 0.514), physical disability (t = 6.343, *p* < 0.001, d = 0.358), psychological disability (t = 5.592, *p* < 0.001, d = 0.315), social disability (t = 5.301, *p* < 0.001,d = 0.304), social handicap (t = 4.452, *p* < 0.001, d = 0.253), and that in the non-clinical populations, females scored significantly higher than males, as did in terms of physical pain (t = 3.055, *p* = 0.002, d = 0.280), psychological discomfort (t = 2.478, *p* = 0.014, d = 0.222), and psychological disability (t = 2.067, *p* = 0.039, d = 0.188).

**Conclusion:**

This study found that the Chinese version of OHIP-14 has measurement invariance between the clinical and non-clinical populations and across genders in the clinical populations, and can be widely used in OHRQoL assessment for public oral investigations.

**Supplementary Information:**

The online version contains supplementary material available at 10.1186/s12903-023-03310-6.

## Background

Nearly 3.5 billion people worldwide have been identified as suffering from oral diseases, according to the Global Burden of Disease Study 2017 [1]. In addition, the China Oral Health Epidemiological Survey 2018 showed the national expenditure on oral diseases was over 29 billion yuan for middle-aged people, with annual growth rates of 11.24% [2]. Oral diseases not only affect patients’ facial, masticatory, and articulatory functions, but also affect their daily life and social activities, causing many negative effects, such as a significant reduction in quality of life, sleep disorders, functional impairment, and psychological disorders [3]. They remain highly prevalent, especially in places where affordable oral care is inaccessible. It was suggested that by educating people about oral disease awareness, prevention, and appropriate management, community oral health would significantly improve [4, 5]. However, front-line clinical workers often focus more on the effects of the oral disease itself but ignore the social, psychological, behavioral, and other oral health-related quality of life (OHRQoL) effects brought about by the disease. This indicated that especially in the environment of tight medical resources, the improvement of oral health education and awareness is weaker, which will further lead to an increased incidence of oral diseases and an increase in social and economic burdens. Therefore, assessing oral health-related quality of life is a task that cannot be neglected by front-line clinical workers in public health dentistry.

OHRQoL is a multidimensional construct that includes physical, social, and psychological dimensions [6] and tries to evaluate how oral health affects every facet of a person’s personal and social life. Specific tools, including the Geriatric Oral Health Assessment Index (GOHAI) [7], Oral Impacts on Daily Performances (OIDP) [8, 9], and the Oral Health Impact Profile (OHIP) [10], were used to measure OHRQoL. The number of items on the scales may vary, and different scales may concentrate on various demographics and dimensions. The OHIP is the most commonly used scale, and its condensed version, known as OHIP-14, has gained widespread acceptance due to its condensed nature and excellent clinical applicability [11, 12]. OHIP-14, for example, can be used in edentulous subjects [13] and responds to 3 dimensions of oral health: functional limitation, pain discomfort, and psychosocial impacts. The inclusion of physical, psychological, social, and physical pain in the 4-factor model was validated in Chinese adults [14], and orthodontic patients [15, 16] also employed it. According to G D Slade’s study, OHIP-14 is a simplified version of OHIP-49 with high reliability and contains questions from each of the seven conceptual dimensions of OHIP-49 [10]. Also, the 7-factor model of OHIP-14 contains functional limitation, physical pain, psychological discomfort, physical disability, psychological disability, social disability, and social handicap, which was adapted from the World Health Organization’s International Classification of Impairments, Disabilities, and Handicaps [17]. Furthermore, the latest research indicated 7-factor of OHIP-14 was developed to address the same concepts as the full version of OHIP but to consume less time [18]. Chinese researchers proved the validity of the 7-factor OHIP-14 model in 2022, and when compared to other factor structure models, the 7-factor model was the most reliable and provided the greatest fit for the current data [19]. Nevertheless, despite the fact that the Chinese version of the OHIP-14 has been extensively used with a variety of people and situations, including the elderly [20], and edentulous subjects [13], it has some limits. Currently, the Chinese version of OHIP-14 is yet to be verified in the clinical populations to see if the 7-dimension is also well suited. Exploring the appropriate structural factors of the OHIP-14 Chinese version would be beneficial to ensuring its use as an important tool for assessing the quality of life in oral health. A deeper investigation is required because the seven dimensions’ application for Chinese clinical patients has not yet been evaluated.

In medicine, cross-cultural validation and psychometric equivalence of a measuring instrument are essential processes to enable its widespread use in international clinical trials [21]. For example, there are scholars addressing the importance of measuring the equivalent of the widely used self-report depression screeners in the population [22]. Similarly, if the OHIP-like measurement results can be improved, it can be better explained that the measurement values are not affected by the latent variables. Moreover, few domestic scholars have done invariance studies on the Chinese version of the OHIP-14 instrument among clinical and non-clinical populations, which is vital to know whether variances in oral health-related quality of life in different populations are due to real health-related differences or to the differences in questionnaire comprehension. Therefore, it is necessary to carry out a measurement invariance study among clinical and non-clinical populations before applying the 7-factor model of the Chinese OHIP scale to clinical and non-clinical groups. Measurement invariance refers to the measurement model invariance of the relationship between observed and latent variables in different aggregates or among different groups in the same aggregate. It is necessary to compare group differences before proceeding [23, 24].

The following models were applied to assess measurement invariance: configural invariance model, determining whether latent variables’ composition is consistent across groups; metric (weak) invariance model, determining whether the factor loadings are consistent across groups; scalar (strong) invariance model, determining whether the intercepts of observed variables are equal among groups; error variance invariance (strict) model, determining whether the error variances are equal among groups. The four models are nested within each other, and the latter step of the test can only be carried out if the requirements of the former step are met [25, 26]. A well-developed measurement invariance test can ensure that the results of the Chinese version of the 7-factor model of the OHIP-14 are indeed differences between different groups, rather than the questionnaire itself causing its results to be biased towards one group, so that the Chinese version of the questionnaire can be more widely used.

In addition, oral illnesses appear to affect men and women differently in terms of their oral health. For instance, the survey revealed that men had worse oral health than women with periodontitis and dental trauma because they were more likely to have harmful behaviors like smoking, drinking alcohol, and chewing betel nuts [27, 28]. However, dental caries and temporomandibular joint pain were more prevalent in women [29, 30], and another study also showed females in pain threshold, time summation, pain expectancy, and body awareness in patients with chronic temporomandibular disease or orofacial pain [31]. The above evidences indicated that gender differences would influence oral health. Furthermore, there were significant differences in the scores of OHIP between different gender groups in a survey of patients with dentofacial deformities in Brazil [32]. Similar results were also seen in the Chinese college student population [19]. Thus, accumulating surveys showed that sex differences may be a significant factor impacting OHRQoL, and it is possible that the latent variable of gender may have an impact on the score of the Chinese version of OHIP-14 in the assessment of individuals of various genders. Therefore, it is also necessary to improve the gender measurement invariance study of OHIP-14.

In summary, this study will explore the 7-factor structure model in clinical populations using validation factor analysis. It will also validate the measurement invariance of the Chinese version of OHIP-14 across groups in clinical and non-clinical populations, as well as the measurement invariance of the Chinese version of OHIP-14 in clinical populations of different genders. The OHIP-14 provides a reliable and valid tool for detailed measurement of the social impact of oral diseases and has potential benefits for clinical decision making and research [10, 33]. It also helps physicians develop a “holistic view” of the social, psychological, and behavioral impacts of oral diseases, which is important for disease prevention and health promotion. Therefore, this study provides a strong scientific basis for the Chinese version of OHIP-14 in assessing oral health-related quality of life and guiding oral surveys in the public.

## Methods

### Participants

The study populations included both the clinical and non-clinical populations. From November 2021 to July 2022, participants for the clinical populations were sought out from the Second Xiangya Hospital of Central South University’s Department of Stomatology. 506 questionnaires were sent out, and 490 valid questionnaires were submitted, with a response rate of 96.8%. All of the patients who took part in the study were able to comprehend the questionnaire’s content and were willing to engage in the survey.

The participants in the non-clinical populations were recruited from October to December 2021 from three universities in Hunan including 212 from Central South University, 302 from Hunan University of Technology and Business, and 424 from Changsha Aviation Vocational and Technical College by convenience sampling by distributing online questionnaires. All participants were informed and willing to cooperate. General descriptions of the participants are shown in Table 1.

**Table 1:** Description of study participants

Variables	Clinical populations		Non-clinical populations	
N	490		919	
Gender	Male	Female	Male	Female
n (%)	240(49.0)	250(51.0)	541(58.9)	378(41.1)
Mean age (SD)	32.69(12.41)	30.63(11.67)	20.40(2.65)	19.88(2.17)

SD: standard deviation

All contributors voluntarily provided written informed consent. Generic data was substituted for information that can be used to identify any specific person. The Human Experiment and Ethics Committee of Second Xiangya Hospital, Central South University, authorized this investigation (KQ2019FY01).

### Instruments

The Chinese version of the OHIP-14 consists of 14 entries and uses a five-point Likert scale with scores ranging from 0 (“never”) to 4 (“very often”), and the total score is negatively correlated with oral health status. The OHIP-14 is the most often used instrument in clinical practice[29], and the Chinese version of the OHIP-14 has been demonstrated to have strong reliability (Cronbach’s alpha (Cronbach’s α) = 0.93) and validity (corrected item-total correlation varied from 0.53 to 0.71)[14].

### Data analysis

The analyses were performed with SPSS 26.0 (IBM SPSS Statistics) and Mplus 8.3. The Chinese version of the OHIP-14 was statistically described (mean, median, standard deviation, minimum, and maximum values), and reliability analysis was performed for each subject using SPSS 26.0. The Kolmogorov-Smirnov test (K-S test) was used to determine normality, and according to Kline’s study, all of the items’ skew and kurtosis coefficients should fall within the acceptable ranges of 3 and 10, respectively [34, 35].

### Item analysis and reliability

The item-total correlations were used to test homogeneity, and the scores above 0.3 were seen as acceptable [36]. The Cronbach’s α was used to evaluate its internal consistency, and results were generally regarded as acceptable when Cronbach’s α was ≥ 0.900 [28].

### Confirmatory factor analysis (CFA)

The Mplus 8.3 software was used to perform CFA and measurement invariance tests on the Chinese version of the OHIP-14. The fit of various factorial models of the Chinese version of OHIP-14 (1-factor model, 3-factor model, 4-factor model, and 7-factor model) was assessed using the CFA. The comparative fit index (CFI), the Tucker-Lewis index (TLI), the standardized root mean square residual (SRMR), and the root mean the square error of approximation (RSMEA), were used to estimate the model fit. The model was considered adequate when CFI ≥ 0.950, TLI ≥ 0.900, RSMEA ≤ 0.060, and SRMR ≤ 0.080[37–39]. The larger the CFI and TLI, the smaller the RSMEA and SRMR, the higher the fitting degree of the modified model. Furthermore, the clinical sample was randomly divided equally into 2 parts using SPSS for cross-validation: the “test group” (n = 233) and “validation group” (n = 257) [18]. This process is applied to evaluate the established model to avoid over-fitting, and CFI needs to be close to 0.950 [37].

### Measurement invariance

A multi-group CFA was conducted to evaluate the measurement invariance of the Chinese version of the OHIP-14 between different samples and across genders in the clinical populations. The study concluded that the measurement invariance was acceptable if the configural invariance model (Model 1), metric (weak) invariance model (Model 2), scalar (strong) invariance model (Model 3), and error variance invariance (strict) model (Model 4) were all valid. The models’ differences in CFI (∆CFI), and RMSEA (∆RMSEA) were used to evaluate the measurement invariance [40]. The invariance model was regarded as acceptable only when ∆CFI < 0.010 and ∆RMSEA < 0.015, and if the change was between 0.010 and 0.020, it indicates that there is a moderate deterioration in the nested model fit, which does not indicate that differences exist. If the change is greater than 0.020, it indicates a definite difference, and the invariance model is rejected [41].

On the assumption that measurement invariance was acceptable, a t-test was performed to examine the statistical significance between different samples and across gender in the clinical populations. We also calculated the effect size by Cohen’s d, which was calculated as the difference of the means of two groups divided by the weighted pooled standard deviations of these groups. Cohen suggested that effect sizes of 0.15-0.40 were small, 0.40 was medium, and ≥ 0.75 were large [42].

## Results

### Descriptive statistics of study subjects

The results of the descriptive statistical analysis of the OHIP-14 Chinese version between the samples are shown in Table 2. In the clinical populations, the mean scores varied from 0.410 to 0.943, with standard deviations varying from 0.715 to 0.956. In the non-clinical populations, the mean scores varied from 0.291 to 0.622, with standard deviations varying from 0.669 to 0.932.

**Table 2:** Descriptive statistics of the responses given to the items of the OHIP-14 by the clinical and non-clinical participants

Item	Mean	Median	Standard Deviation	Maximum	Minimum	Kurtosis	Skewness
1	0.410/0.397	0/0	0.715/0.783	4/4	0/0	3.305/5.590	1.830/2.330
2	0.500/0.364	0/0	0.829/0.754	4/4	0/0	2.602/5.972	1.716/2.399
3	0.943/0.622	1/0	0.946/0.932	4/4	0/0	-0.165/1.240	0.680/1.413
4	0.916/0.554	1/0	0.949/0.888	4/4	0/0	-0.124/2.007	0.728/1.601
5	0.663/0.305	0/0	0.936/0.755	4/4	0/0	1.065/7.572	1.316/2.779
6	0.757/0.322	0/0	0.950/0.768	4/4	0/0	0.854/7.134	1.161/2.679
7	0.931/0.616	1/0	0.956/0.885	4/4	0/0	-0.386/0.706	0.646/1.265
8	0.712/0.457	0/0	0.904/0.821	4/4	0/0	0.418/3.605	1.065/1.952
9	0.727/0.464	0/0	0.885/0.811	4/4	0/0	0.315/3.159	1.007/1.858
10	0.633/0.447	0/0	0.862/0.810	4/4	0/0	1.262/3.468	1.303/1.931
11	0.539/0.326	0/0	0.815/0.697	4/4	0/0	1.781/5.665	1.484/2.365
12	0.510/0.320	0/0	0.770/0.703	4/4	0/0	1.660/6.591	1.446/2.517
13	0.600/0.448	0/0	0.835/0.831	4/4	0/0	1.315/4.049	1.306/2.052

Insert “/” between the statistical values to separate the statistical values of different populations. “the clinical populations / the non-clinical populations”

In addition, the absolute values of the skewness coefficients for each item varied from 0.646 to 1.830, and the absolute values of the kurtosis coefficients varied from 0.124 to 3.305 in the clinical populations. The absolute values of the skewness coefficients for the non-clinical populations varied from 1.265 to 2.779, and the absolute values of the kurtosis coefficients varied from 0.706 to 7.709. The K-S test was used to determine normality, and all of the items’ skew and kurtosis coefficients should fall within the acceptable ranges of 3 and 10, respectively[34, 35]. Therefore, the distribution of each item was close to normality.

### Confirmatory factor analysis

Before measuring measurement invariance, CFA was used to see how well the Chinese version of OHIP-14 fit different factorial models (1-factor model, 3-factor model, 4-factor model, and 7-factor model) in clinical populations. The results are shown in Table 3. The model fit was estimated using CFI, TLI, SRMR, and RSMEA, and the models were considered to be adequate when CFI ≥ 0.950, TLI ≥ 0.900, RSMEA ≤ 0.060, and SRMR ≤ 0.080. Comparing with other factorial models, the 7-factor model (CFI = 0.970, TLI = 0.952; SRMR = 0.029, RMSEA = 0.052(0.040,0.063)) fit to all subjects excellently and adequately. The results of cross-validation are shown in Supplemental Table 1. The result of the test group in terms of the 7-factor model showed a good fit (CFI = 0.970; TLI = 0.951; SRMR = 0.035, RMSEA = 0.053(0.032,0.071)). Also, the validation group showed valid fit results (CFI = 0.969, TLI = 0.950, SRMR = 0.031, RMSEA = 0.057(0.039, 0.074)).

**Table 3:** The fit of factorial model of the Chinese version of OHIP-14 in the clinical populations

Model	*χ* ^*2*^	*df*	CFI	TLI	SRMR	RMSEA
Unifactorial	454.078	77	0.848	0.820	0.061	0.100(0.091,0.109)
3 Factors-1st Order	272.286	74	0.920	0.901	0.048	0.074(0.065,0.083)
4 Factors-1st Order	212.470	71	0.943	0.927	0.055	0.064(0.054,0.074)
7 Factors-1st Order	129.044	56	0.970	0.952	0.029	0.052(0.040,0.063)

*χ2*, chi-square; *df*, degree of freedom; CFI, comparative fit index; TLI, Tucker-Lewis index; SRMR, standardized root mean square residual; RMSEA, root-mean-square error of approximation

### Reliability

In the clinical populations, the item-total correlations between factors varied from 0.343 to 0.749, and the item-total correlations varied from 0.561 to 0.821. In the non-clinical populations, item-total correlations between factors varied from 0.479 to 0.821, and the item-total correlations varied from 0.759 to 0.862. The item-total correlations between factors and the item-total correlations in both the clinical and non-clinical populations were greater than 0.3, indicating that the homogeneity was adequate.

The total Cronbach’s α coefficient in the clinical populations was 0.942, and the Cronbach’s α of 7 factors was as follows: functional limitation (items 1 and 2) 0.672; physical pain (items 3 and 4) 0.846; psychological discomfort (items 5 and 6) 0.802; physical disability (items 7 and 8) 0.849; psychological disability (items 9 and 10) 0.728; social disability (items 11 and 12) 0.849; social handicap (items 13 and 14) 0.854. In the non-clinical populations, the total Cronbach’s α was 0.958, and the alphas for each of the seven factors were as follows: functional limitation 0.800; physical pain 0.854; psychological discomfort 0.902; physical disability 0.850; psychological disability 0.768; social disability 0.862; and social handicap 0.819.

### Measurement invariance

The results of the measurement invariance between different samples are shown in Table 4. The 7-factor model configural invariance model was considered adequate (CFI ≥ 0.900, TLI ≥ 0.900, SRMR ≤ 0.080, RSMEA ≤ 0.080). The metric (weak) invariance model showed a valid fit (∆CFI and ∆RMSEA were both less than 0.015). Given that support, the next step of the analysis could be performed. The scalar (strong) invariance model met the standard for invariance parameters (∆CFI and ∆RMSEA were both less than 0.015). In the final error variance invariance (strict) model, ∆RMSEA was less than 0.015, while ∆CFI was greater than 0.015. There is a moderate deterioration in the model fit, which does not indicate the existence of differences, so strict invariance is also acceptable.

**Table 4:** Measurement invariance model between different samples fitting indices and comparison

Model	*χ² (df)*, *p*	CFI	TLI	SRMR	RMSEA	Model comparison	∆CFI	∆RMSEA	
The clinical populations	129.044(56), *p* < 0.001	0.970	0.952	0.029	0.052				
The non-clinical populations	102.076(56), *p* < 0.001	0.987	0.978	0.023	0.030				
Model 1	229.067(112), *p* < 0.001	0.981	0.969	0.025	0.039				
Model 2	242.511(119), *p* < 0.001	0.980	0.970	0.029	0.038	2 vs. 1	-0.001		-0.001
Model 3	259.125(126), *p* < 0.001	0.979	0.969	0.030	0.039	3 vs. 2	-0.001		0.001
Model 4	375.653(140), *p* < 0.001	0.962	0.951	0.038	0.049	4 vs. 3	-0.017		0.010

Model 1 = configural invariance; Model 2 = metric invariance; Model 3 = scalar invariance; Model 4 = error variance invariance; *χ²*, chi-square; *df*, degree of freedom; CFI, comparative fit index; TLI, Tucker-Lewis index; SRMR, standardized root mean square residual; RMSEA, root-mean-square error of approximation; ∆, change in the parameter

Similarly, as shown in Table 5, the configural, metric, scalar, and error variance invariance were all found to be validated by the data when tested across gender in the clinical populations, confirming that the measurement invariance of the Chinese version of the OHIP-14 also holds across gender in patients,, with each model fit index reaching ∆CFI < 0.015 and ∆RMSEA < 0.015.

**Table 5:** Measurement invariance model across genders in the clinical populations fitting indices and comparison

Model	*χ²(df), p*	CFI	TLI	SRMR	RMSEA	Model comparison	∆CFI	∆RMSEA		
Male	132.008(56), *p* < 0.001	0.951	0.920	0.042	0.074					
Female	99.021(56), *p* < 0.001	0.965	0.943	0.032	0.057					
Model 1	230.333(112), *p* < 0.001	0.957	0.930	0.037	0.066					
Model 2	237.064(119), *p* < 0.001	0.957	0.934	0.039	0.064	2 vs. 1	0.000			-0.002
Model 3	249.092(126), *p* < 0.001	0.955	0.935	0.040	0.063	3 vs. 2	-0.002			-0.001
Model 4	264.613(140), *p* < 0.001	0.955	0.941	0.049	0.060	4 vs. 3	0.000			-0.003

Model 1 = configural invariance; Model 2 = metric invariance; Model 3 = scalar invariance; Model 4 = error variance invariance; *χ²*, chi-square; *df*, degree of freedom; CFI, comparative fit index; TLI, Tucker-Lewis index; SRMR, standardized root mean square residual; RMSEA, root-mean-square error of approximation; ∆, change in the parameter

### Comparison of OHIP-14 Chinese version scores of different cohorts

The t-test for scores of the Chinese version of the OHIP-14 between the participants is shown in Table 6. The clinical populations tend to have higher general scores than the non-clinical populations (t = 7.046, *p* < 0.001, d = 0.369). In addition, the clinical populations scored higher than the non-clinical populations in terms of functional limitation (t = 2.178, *p* = 0.030, d = 0.125), and significantly higher than the non-clinical populations in terms of physical pain (t = 7.880, *p* < 0.001,d = 0.436), psychological discomfort (t = 8.993, *p* < 0.001, d = 0.514), physical disability (t = 6.343, *p* < 0.001, d = 0.358), psychological disability (t = 5.592, *p* < 0.001, d = 0.315), social disability (t = 5.301, *p* < 0.001,d = 0.304), and social handicap (t = 4.452, *p* < 0.001, d = 0.253).

**Table 6:** Measurement invariance model between different samples fitting indices and comparison

	Mean (SD)		t	*p*	Cohen’s d
	Clinical populations (n = 490)	Non-clinical populations (n = 919)			
Functional limitation	0.91(1.345)	0.74(1.371)	2.178*	0.030	0.125
Physical pain	1.86(1.766)	1.12(1.629)	7.880**	< 0.001	0.436
Psychological discomfort	1.42(1.725)	0.61(1.412)	8.993**	< 0.001	0.514
Physical disability	1.64(1.728)	1.05(1.567)	6.343**	< 0.001	0.358
Psychological disability	1.36(1.550)	0.89(1.432)	5.592**	< 0.001	0.315
Social disability	1.05(1.480)	0.63(1.278)	5.301**	< 0.001	0.304
Social handicap	1.07(1.492)	0.71(1.351)	4.452**	< 0.001	0.253
OHIP-14 TOTAL	9.31(9.190)	5.75(8.799)	7.046**	< 0.001	0.396

SD: standard deviation; *p < 0.05 (two tailed), **p < 0.01 (two tailed)

The t-test for scores of the OHIP-14 model across genders in the clinical populations is shown in Table 7. Female patients had higher mean values in the total scores than males, but they were not statistically different (t = 1.952, *p* = 0.052, d = 0.176). On each item, female patients also scored higher than male patients in psychological discomfort (t = 2.478, *p* = 0.014, d = 0.222) and psychological disability (t = 2.067, *p* = 0.039, d = 0.188) and significantly higher than male patients on physical pain (t = 3.055, *p* = 0.002, d = 0.280).

**Table 7:** Measurement invariance model across genders fitting indices and comparison

	**Mean (SD)**		t	*p*	Cohen’s d
	**Female (n = 250)**	Male (n = 240)			
Functional limitation	0.94(1.391)	0.88(1.296)	0.500	0.617	0.045
Physical pain	2.10(1.862)	1.61(1.627)	3.055**	0.002	0.280
Psychological discomfort	1.61(1.854)	1.23(1.560)	2.478*	0.014	0.222
Physical disability	1.76(1.760)	1.52(1.689)	1.586	0.113	0.139
Psychological disability	1.50(1.677)	1.21(1.394)	2.067*	0.039	0.188
Social disability	1.12(1.564)	0.97(1.386)	1.149	0.251	0.102
Social handicap	1.07(1.417)	1.07(1.517)	0.009	0.993	0.000
OHIP-14 TOTAL	10.10(9.623)	8.49(8.660)	1.952	0.052	0.176

SD: standard deviation; *p < 0.05 (two tailed), **p < 0.01 (two *tailed)*

## Discussion

The CFA of the clinical populations showed that the 7-factor model of the Chinese version of the OHIP-14 had the best fit among the factor models, indicating that the 7-factor model can better reflect the quality of life of Chinese clinical patients in terms of oral health. Also, this study examined the measurement invariance between the clinical and non-clinical populations and across gender in the clinical populations of the Chinese version of the OHIP-14. Overall, the Chinese version of the 7-factor model of OHIP can be well applied to Chinese populations and widely used in OHRQoL assessment for public oral investigations.

The CFA results demonstrated that the 7-factor scale of the OHIP-14 has a better fit for the clinical populations. The same results were also found in the Chinese version of OHIP-14 tested on Chinese college students [19], indicating that the 7-factor structure of the Chinese version of OHIP-14 can be well applied to both clinical and non-clinical populations. According to the G D Slade’s study, OHIP-14 is a simplified version of OHIP-49 with high reliability and contains questions from each of the seven conceptual dimensions of the OHIP-49 [10]. Also, the latest research indicated 7-factor of OHIP-14 were developed to address the same concepts as the full version of OHIP but to consume less time [18]. Based on the result of CFA, we performed a measurement equivalence test for the 7-factor model of OHIP-14.

To make the scale more suitable for clinical research, it is necessary to conduct measurement equivalence research. Measurement equivalence is an important dimension to reflect the quality and stability of questionnaire tools and is an important prerequisite for the comparison of differences between groups [24]. Between-group differences are valid and interpretable only if they meet the requirements of measurement equivalence. For example, scholars are addressing the importance of measuring the equivalent of the widely used self-report depression screeners in the population[22]. Similarly, if the OHIP-like measurement results can be improved, it can be better explained that the measurement values are not affected by the latent variables (age, gender, clinical status). Previous studies have validated measurement equivalence between sexes in college students [19], but their conclusions have been limited to nonclinical populations. Therefore, this study extends it to clinical populations and between clinical populations, as well as between different genders within clinical populations. The results of multi-group CFA showed that the configural, metric, scalar, and error variance invariance were supported by the survey data, indicating that the scores are meaningfully comparable between groups and similarly significant for clinical and non-clinical populations, as well as across gender in clinical populations. It is worth noting that there was a moderate deterioration in model fit in the strict invariance test between the clinical and non-clinical populations, but this does not preclude the validity of the invariance test [40]. The results of the measurement invariance test indicate that the 7-factor OHIP-14 scale holds both between the clinical and non-clinical populations and across genders in the clinical populations, suggesting that it can be widely used with the Chinese populations.

Based on measurement invariance, the T-test was performed to compare scores between the clinical and non-clinical populations. The results found that the clinical populations had significantly higher scores than the non-clinical populations on the Chinese version of the OHIP-14 in terms of total scores and on all factors. Also, except for the functional limitation, other factors had small or middle effect size between different groups. This is because the clinical populations have a higher proportion of positive disease, and oral disease has a more significant impact on their quality of life, including but not limited to dental caries, endodontic inflammation, periodontal disease, and temporomandibular joint disorders. These disorders often lead to difficulty in articulation, pain, discomfort when eating, emotional tension, difficulty relaxing, irritability, social fatigue, an inability to perform daily tasks, reduced satisfaction with life, etc. This in turn is reflected in the scores on the various dimensions of the 7-factor OHIP-14 scale, resulting in even higher scores. The psychometric equivalence of OHIP-14 shows that it can be widely applied in both clinical and non-clinical populations.

Also, in a t-test across genders in the clinical populations, the results did not show significant differences in the total scores. This suggests that in the same state of oral disease, there is no difference in the degree of oral distress between men and women. In future studies, even if there is a gender difference in prevalence, the sample size should be increased to reasonably evaluate the difference in scores or the Euler error. But was further evaluated in dimension, male patients showed significantly lower scores than female patients on the three dimensions: physical pain, psychological discomfort, and psychological disability, which also had a small effect size. However, this difference was even more significant in the non-clinical populations [19]. In the non-clinical populations, the t-test for scores showed that females scored significantly higher than men as did in terms of physical pain, psychological discomfort, and psychological disability. This suggests that although there is no significant difference in the overall quality of life, the OHIP-14 can provide doctors with a detailed quality of life assessment under the 7 dimensions of the assessment. This result may be due to the greater sensitivity of women to physiological pain in previous studies [43, 44], and a meta-analysis showed that women were more sensitive than men, with mean effect sizes ranging from d = 0.09 to 0.82 [45]. Similarly, data from routine dental examinations by the Swedish Public Dental Care Service show that women have a significantly higher rate of maxillofacial pain than men [46]. This can also be reflected in low scores for psychological discomfort and psychological disability. This suggests that the differences in scores on the Chinese version of the OHIP-14 are a true reflection of the differences in quality of life in oral health between clinical populations and are not due to measurement inequalities in the questionnaire instrument.

The rise in oral disease burden in countries is indeed becoming a matter of concern and is related to social and economic changes. Our study performed the 7-factor construct validity and measurement equivalent across clinical status and genders, which is an important assessment of oral disease burden. Thanks to the minimal burden to subjects and individuals collecting the data [47], the use of this validated OHIP-14 provides an opportunity to perform OHRQoL assessment in almost any setting, which could effectively assess the urgent need to address oral diseases among others as a global health priority for a clinical investigation [48].

Some limitations of this research should be focused on in future studies. The university students were used as the non-clinical populations for the cross-clinical invariance measures in this study, which resulted in an incomplete match with the clinical populations in terms of demographic information such as age distribution and educational attainment. In the future, further research should be conducted using populations that are more demographically compatible with the clinical populations.

## Conclusions

In summary, it can be demonstrated that the 7-factor model of the Chinese version of the OHIP-14 scale can be used as a reliable, simple and effective tool for large-scale screening of oral health quality of life.

### Electronic supplementary material

Below is the link to the electronic supplementary material.

Supplementary Material 1

## Data Availability

The datasets generated and/or analysed during the current study are not publicly available due to privacy reasons, but are available from the corresponding author on reasonable request.

## List of Abbreviations

OHRQoL: Oral health-related quality of life
OHIP: Oral Health Impact Profile
CFA: Confirmatory factor analysis
GOHAI: The Geriatric Oral Health Assessment Index
OIDP: Oral Impacts on Daily Performances
Cronbach’s α: Cronbach’s alpha coefficient
K-S test: The Kolmogorov-Smirnov test
CFI: The comparative fit index
TLI: The Tucker–Lewis index
SRMR: The standardized root mean square residual
RSMEA: The root means the square error of approximation
∆CFI: The models’ differences in CFI
∆RMSEA: The models’ differences in RMSEA
