# Supplementary material for: Factorial structure and measurement invariance of the Chinese version of the Oral Health Impact Profile-14 among clinical populations and non-clinical populations: an evidence for public oral investigations
Source: BMC Oral Health. 2023 Aug 24;23:588. doi: 10.1186/s12903-023-03310-6 (PMC10463897; doi:10.1186/s12903-023-03310-6)
Supplement: Supplementary file 1 — Supplementary Material 1 [file 12903_2023_3310_MOESM1_ESM.docx]

**Supplemental Table 1**. goodness of fit indices of the confirmatory factor analysis in the test group ans validation group.

| **Samples** | ***N*** | ***χ^2^*** | ***df*** | **CFI** | **TLI** | **SRMR** | **RMSEA** |
| --- | --- | --- | --- | --- | --- | --- | --- |
| Total | 490 |  |  |  |  |  |  |
| Test | 233 | 92.098 | 56 | 0.970 | 0.951 | 0.035 | 0.053(0.032,0.071) |
| Validation | 257 | 102.023 | 56 | 0.969 | 0.950 | 0.031 | 0.057(0.039,0.074) |

*χ2*, chi-square; *df*, degree of freedom; CFI, comparative fit index; TLI, Tucker-Lewis index; SRMR, standardized root mean square residual; RMSEA, root-mean-square error of approximation;
